# AVL9 promotes colorectal carcinoma cell migration via regulating EGFR expression

**DOI:** 10.1186/s12575-021-00162-8

**Published:** 2022-01-06

**Authors:** Qiong Wu, Jing De Chen, Zhuqing Zhou

**Affiliations:** 1 Department of Oncology, Shanghai East Hospital, School of Medicine, Tongji University, Shanghai, 200120 China; 2 Department of Gastrointestinal Surgery, School of Medicine, Tongji University, Shanghai, 200120 China

**Keywords:** AVL9, Colorectal cancer, Metastasis, EGFR

## Abstract

**Background:**

Despite advanced treatments could inhibit progression of colorectal carcinoma (CRC), the recurrence and metastasis remain challenging issues. Accumulating evidences implicated that AVL9 played a vital role in human cancers, but it’s biological function and mechanism in CRC remain unclear.

**Aim:**

To investigate the biological role and mechanism of AVL9 in colorectal carcinoma.

**Results:**

AVL9 expression was significantly upregulated in tumor tissues than that in matched normal tissues both at mRNA and protein levels. High expression of AVL9 was closely correlated with M status, stages and poor prognosis of colorectal carcinoma (CRC) patients. Functionally, AVL9 overexpression promoted cell migration rather than cell proliferation in vitro, whereas AVL9 knockdown exhibited the contrary results. Mechanistically, AVL9 regulated EGFR expression, and knockdown of EGFR restrained AVL9-induced cell migration.

**Conclusion:**

These findings demonstrated that AVL9 contributed to CRC cell migration by regulating EGFR expression, suggesting a potential biomarker and treatment target for CRC.

## Background

Colorectal carcinoma (CRC), ranking as one of the most common cancers among males and females, is an important threat to human health [1]. The incidence of colorectal cancer is second only to lung cancer and breast cancer [2]. CRC is a multifactorial disease that can be caused by risk factors such as age, obesity, smoking habit, alcohol use and family history [3]. Intrusive procedures, such as colonoscopy, are the primary diagnostic means [4]. With progression of the disease, non-visible micro-metastases may occur while no obvious symptoms in the early stage [5]. Clinical limitations, specifically, resection evasion during surgery and insufficiently treatment with chemotherapy, lead to a low 5-year survival rate [6, 7]. Therefore, it’s urgent to provide a novel biomarker for CRC patients.

AVL9, an exocytosis gene in yeast [8], was first defined to be involved in cell polarity and cell migration [9]. Notably, previous studies suggested that AVL9 was crucially important to tumorigenesis. For instance, Liang et al reported that AVL9 may be served as a direct target of miR-203a-3p in non-small-cell lung cancer (NSCLC). The functional assays indicated that overexpressed miR-203a-3p suppressed cell proliferation, migration and invasion by targeting AVL9 in NSCLC cells [10]. In addition, CRPAT4, hypoxia-regulated IncRNA, worked as an oncogene in cell proliferation and migration via facilitating AVL9 translation in clear cell renal cell carcinomas (ccRCC) [11]. What’s more, AVL9 was confirmed as a direct target of miR-497-5p in CRC. And mechanistic studies showed that downregulated Linc00662 significantly suppressed CRC progression and metastasis attributed to compete with miR-497-5p to modulate the expression of AVL9 [12]. Nevertheless, the specific role of AVL9 in CRC is still unknown.

In current study, data from the cancer genome atlas (TCGA) database showed that expression of AVL9 was observably increased in cancer tissue compared to non-cancer tissue, as was consistent with our results of IHC analysis. Interestingly, biological experiments demonstrated that AVL9 played a vital effect on CRC cell metastasis, but not on proliferation. Moreover, mechanistic researches revealed that AVL9 promoted CRC cell migration via regulating EGFR expression, implying that AVL9 has the potential to be a biomarker for early diagnosis and a novel therapeutic target.

## Results

### AVL9 is upregulated in colorectal carcinoma

**Fig. 1:**
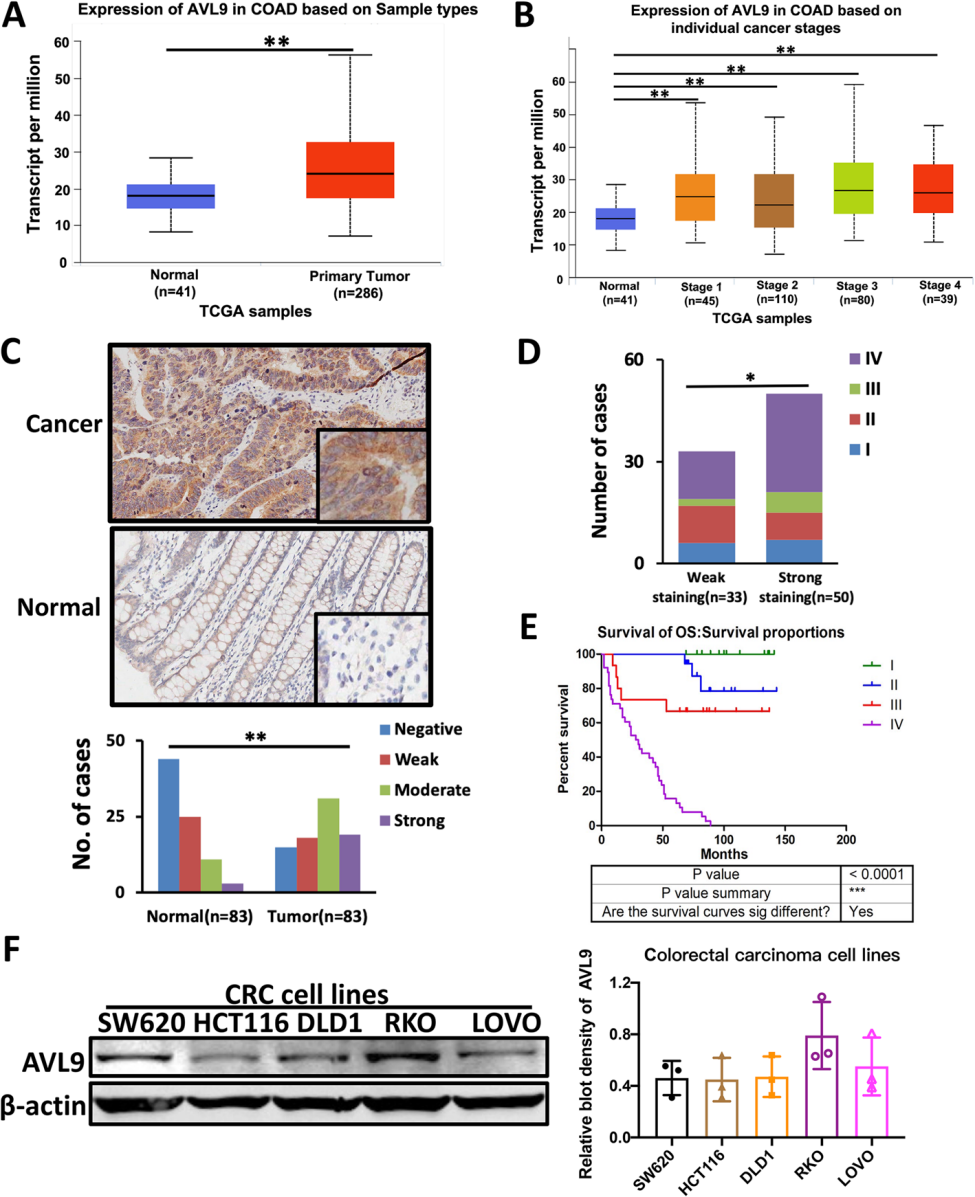
AVL9 was aberrantly elevated in colorectal carcinoma. **A** The expression of AVL9 at mRNA level in CRC tissue was obtained from TCGA database. **B** AVL9 expression was positive correlation with stages of CRC tissue. **C** Representative pictures of CRC samples with IHC staining was shown. **D** Statistical analysis of AVL9 staining strength in a tissue microarray containing 83 pairs of CRC samples. *Chi*-square test was used. **E** Overall survival rate was shown at different stages of CRC patients. **F** Relative AVL9 expression at protein level in CRC cell lines was determined by western blot analysis. The bar graph showed the relative intensity of AVL9 protein normalized by β-actin. Data are means ± SEM (*n* = 3). *P<0.05, **P<0.01, ***P<0.001

To investigate the role of AVL9 in CRC, we analyzed AVL9 expression at mRNA level in colorectal carcinoma based on TCGA database. The results showed that AVL9 expression was substantially higher in cancer tissues (*n*=286) than that in normal tissues (*n*=41) (Fig. 1A, *P*<0.05). Meanwhile, in accordance with clinical data from TCGA database, expression of AVL9 increased gradually from earlier stage to advanced stage of CRC (Fig. 1B, *P*<0.05). Next, in order to detect AVL9 expression at protein level, we analyzed the tissue microarray containing 83 tumor samples and 83 non-tumor samples. Consistent with the above bioinformatics, increased protein level of AVL9 expression in CRC tumor tissues than that in the paired normal tissues was observed by IHC staining. AVL9 expression was upregulated 50/83 (60.2%) in CRC samples (*n*=83) on the basis of classification of staining results as mentioned before (Fig. 1C). Among them, advanced stages (III + IV) showed strong staining, however, earlier stage (I + II) showed weak staining, indicating that AVL9 expression was positive correlation with stage of CRC (Fig. 1D, *P*<0.05). Similarly, higher expression of AVL9 was distinctly related to M status (*P* = 0.001) and stages (*P* = 0.048), rather than other clinicopathological characteristic, such as gender, age, tumor size, differentiation, T classification and N status (Table 1). Moreover, compared to lower AVL9 expression at earlier stages (I + II), patients with higher AVL9 expression at advanced stages (III + IV) showed a significantly lower overall survival rate (Fig. 1E). Collectively, data showed above reveals that AVL9 is upregulated in CRC samples and high expression was associated with CRC progression.

**Table 1:** The correlation of AVL9 expression with clinicopathological features of colorectal carcinoma patients

	Number of cases	Negative, weak	Moderate, strong	*P* value
	(*n* = 83)	(*n* = 50)	(*n* = 33)	
Age (years)				
> 50	60	33	27	0.115
≤ 50	23	17	6	
Gender				
Male	50	27	23	0.152
Female	33	23	10	
Tumor size				
≤ 5 cm	50	28	22	0.331
> 5 cm	33	22	11	
T classification				
T1, T2	17	12	5	0.328
T3, T4	66	38	28	
N status				
N0	52	29	23	0.281
N1-3	31	21	10	
Metastasis				
M0	35	14	21	0.001
M1	48	36	12	
Stage				
I, II	32	15	17	0.048
III, IV	51	35	16	

### AVL9 enhances the CRC cell migration in vitro

**Fig. 2:**
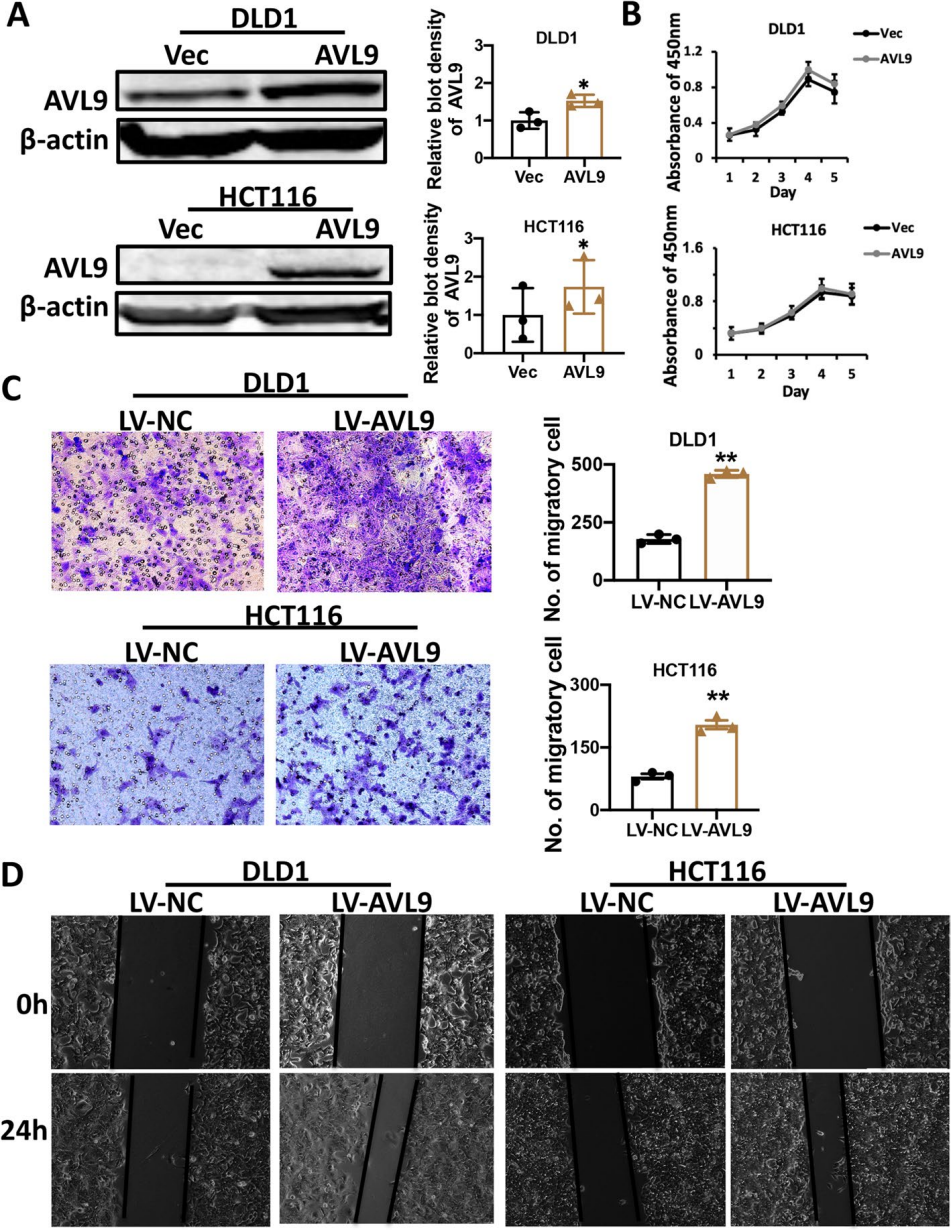
Effects of AVL9 overexpression on CRC cells proliferation and migration in vitro. Notes: DLD1 and HCT116 cells were transfected with plasmids for 24h respectively. **A** The overexpression efficacy of AVL9 was verified by western blot assay. The bar graph showed the relative intensity of AVL9 protein normalized by β-actin. **B** CCK-8 assay showed that no remarked change was observed following AVL9 overexpression in DLD1 and HCT116 cells; however, AVL9 overexpression significantly promoted cell migration which were determined by Trans-well assay (**C**) and Wound healing assay (**D**). All the experiments were performed at least three times. All values were represented as means ± SEM. *P<0.05

In order to further explore biological function of AVL9 in CRC progression, we detected AVL9 expression at protein level in five kinds of CRC cell lines by western blot assay (Fig. 1F). Three kinds of CRC cell lines (DLD1, HCT116 and RKO) were selected for subsequent studies. DLD1 and HCT116, low-expressed AVL9, were overexpressed by transiently transfecting with AVL9 overexpressed plasmid. Transfection efficiency was validated by western blot assay (Fig. 2A). The proliferation assay of DLD1 cell transfected with AVL9 plasmid showed no significantly change of growth rates than the control group, which is consistent in HCT116 cell (Fig. 2B). To examine the effect of AVL9 overexpression on the cell migratory ability, DLD1 and HCT116 cell stably overexpressing AVL9 were established and then trans-well assay was carried out. The results indicated that AVL9 could markedly increase the DLD1 and HCT116 cell migration (Fig. 2C). Subsequently, scratch-wound healing assay was performed. Cells overexpressed AVL9 showed stronger wound healing ability compared to control group, suggesting a positive impact of AVL9 on CRC cell migration (Fig. 2D). These findings collectively implicate that AVL9 facilitates the CRC cell migration in vitro.

### Knockdown of AVL9 significantly inhibits CRC cell migration in vitro

**Fig. 3:**
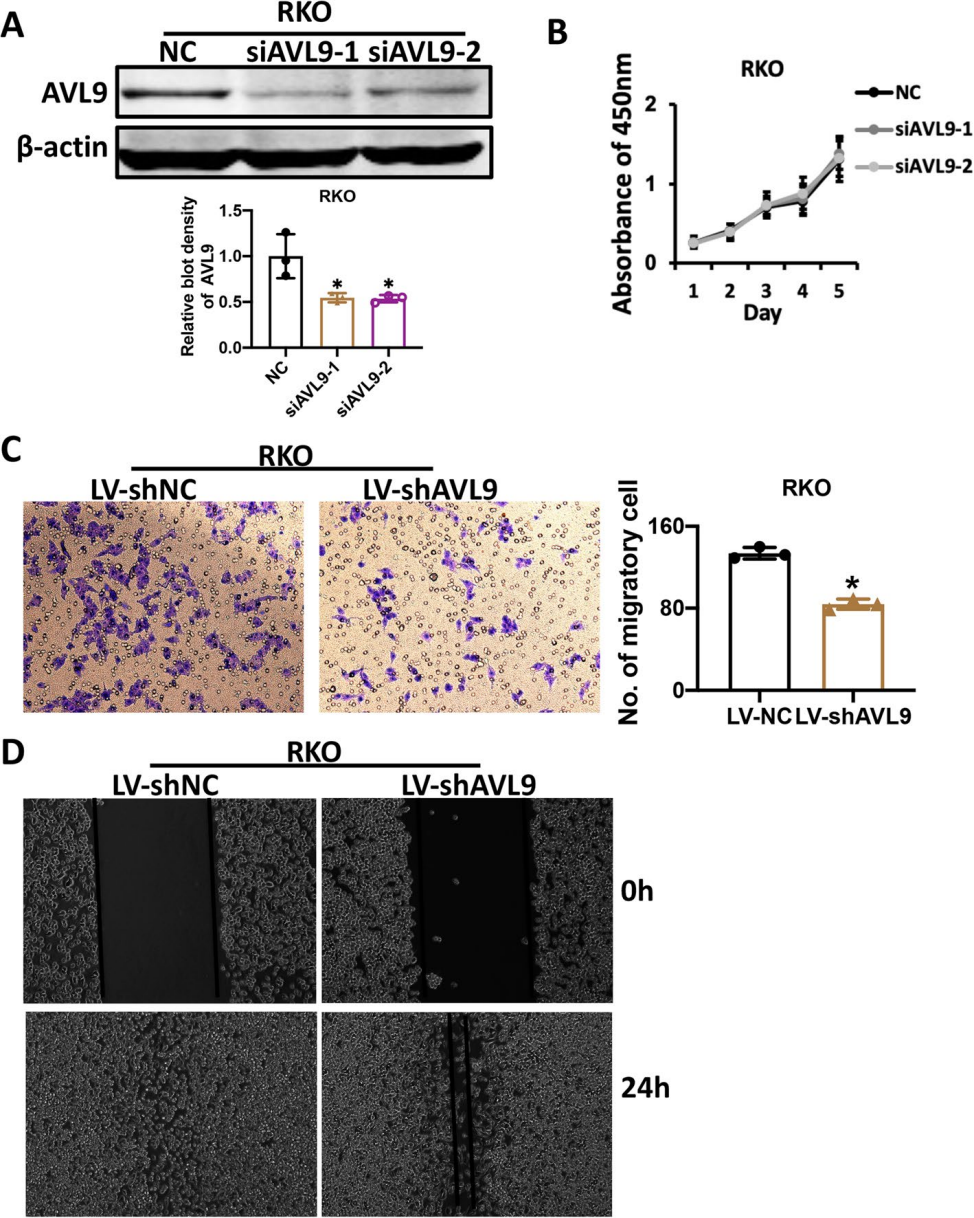
AVL9 knockdown inhibited RKO cell migration. AVL9 expression was downregulated in RKO cell by transfecting specific siRNA (siAVL9-1/siAVL9-2) which was confirmed by western blot assay. The bar graph showed the relative intensity of AVL9 protein normalized by β-actin (**A**). Effect of AVL9 knockdown on cell growth was detected by CCK-8 assay (**B**). AVL9 silencing decreased the migration of RKO cell verified by Trans-well assay (**C**) and Wound healing assay (**D**). Data was represented as means ± SEM. of three replicates. *P<0.05

Next, loss-of-function experiments were carried out to explore the role of AVL9 in RKO cell line. Two AVL9-specific siRNA1, siRNA2 and NC siRNA were constructed and respectively transfected in the RKO cell. Transfection efficacy was confirmed by western blot analysis (Fig. 3A). Cell counting for five consecutive days (CCK-8 assay) was performed to assess the potential effects upon downregulating AVL9 expression on cell proliferation. As is shown in Fig. 3B, no remarkable changes were observed. Trans-well assay demonstrated that stably silencing AVL9 in RKO cell significantly inhibited the cell migration compared with the control group (Fig. 3C). In addition, wound healing assay, measurements of cell motility after 24h, revealed that AVL9 knockdown could restrain the migration of RKO cell (Fig. 3D). These data further confirm that ALV9 performs a crucial role in CRC cell migration, rather than cell proliferation.

### AVL9 regulates EGFR expression in CRC cells

**Fig. 4:**
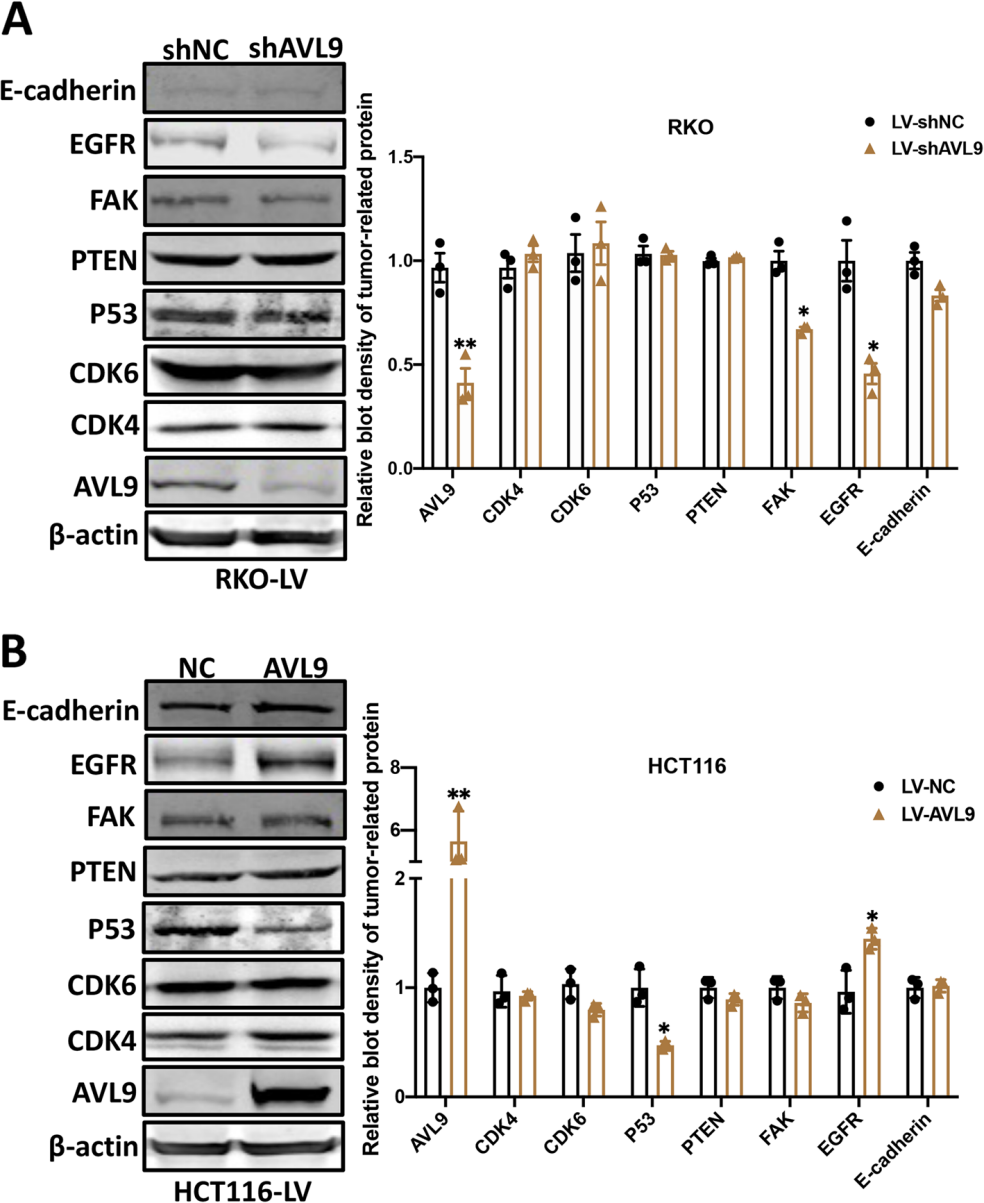
AVL9 upregulated EGFR protein level expression. **A** Western blot analysis confirmed the effect of AVL9 on tumor-related factors in RKO-Lv3-shAVL9/ RKO-Lv3-shNC and HCT116-Lv6-AVL9/HCT116-Lv6-NC cells. Silencing AVL9 reduced the expression of EGFR at protein level in RKO-Lv3-shAVL9 cell compared to RKO-Lv3-shNC, simultaneously, AVL9 overexpression upregulated the protein level of EGFR expression in HCT116-Lv6-AVL9 compared to the control group (**B**). The bar graph showed the relative intensity of tumor-related protein normalized by β-actin *P<0.05

**Fig. 5:**
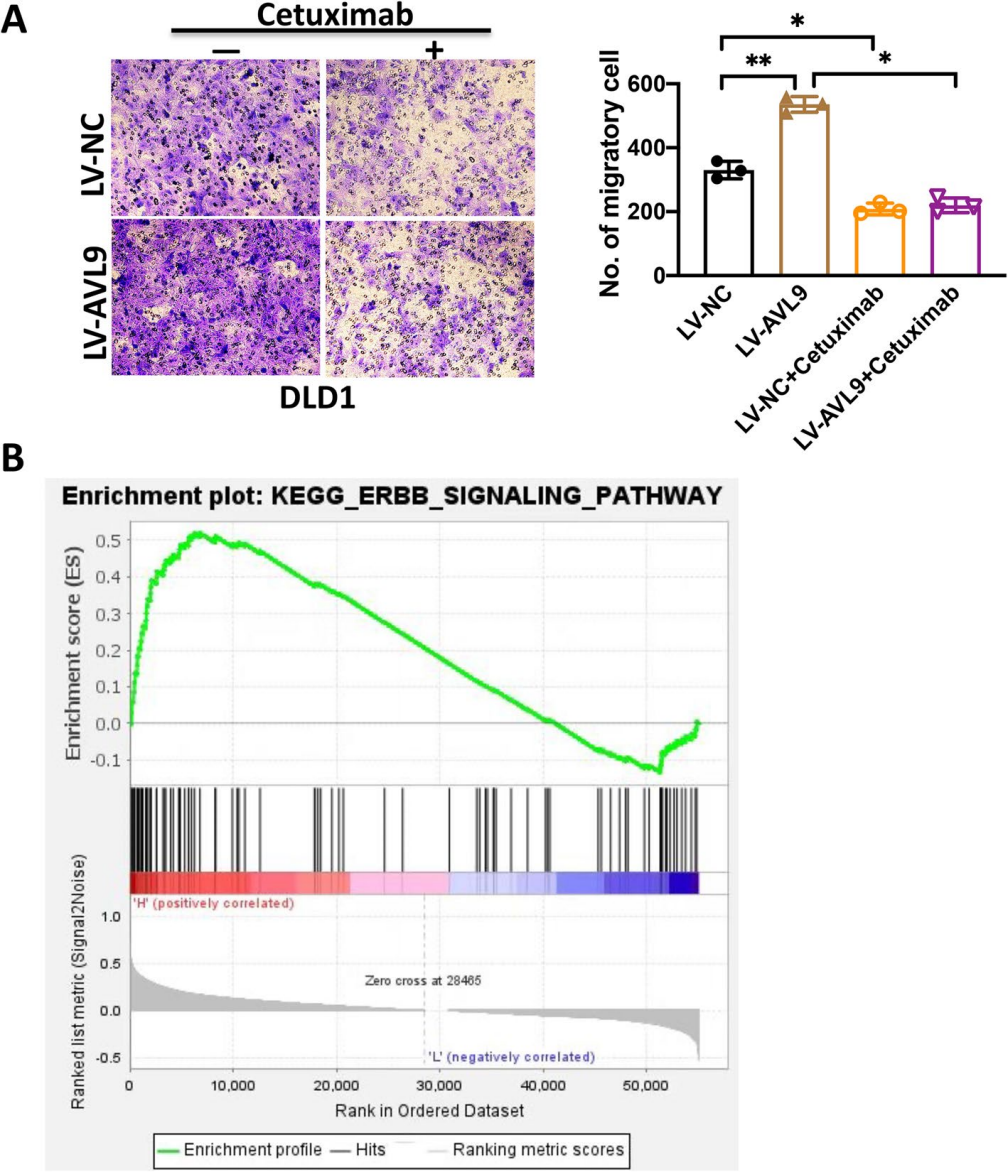
AVL9 promoted CRC cell migration via regulating EGFR expression. **A** Rescue experiment showed that decreased EGFR expression could partially suppressed the increased migration in DLD1-Lv6-AVL9 compared to DLD1-Lv6-NC cell. Cells were treated or not with concentrations of cetuximab (1μM). **B** GSEA showed that ERBB signaling pathway was upregulated in AVL9 high expression group. The CRC patients were categorized into high expression group and low expression group by mean expression of AVL9. *P<0.05, **P<0.01

To investigate the molecular mechanism of how AVL9 promotes CRC cells migration, western blot analysis was performed to examine the expression of tumor proliferation- and metastasis-related proteins, including E-cadherin, EGFR, FAK, PTEN, P53, CDK4 and CDK6. As is shown in Fig. 4A, EGFR expression at protein level decreased following AVL9 knockdown in RKO cell. Consistent and significant conclusion were obtained through AVL9 overexpression in HCT116 cell (Fig. 4B). Epidermal Growth Factor Receptor (EGFR), correlated positively with cancers progression and poor prognosis, was reported highly expressed in different types of tumors [13, 14]. Thus, we supposed that AVL9 promotes cell migration mainly via regulating EGFR expression in CRC. For patients with metastatic CRC (mCRC), cetuximab (CTX), as an EGFR monoclonal antibody, is a typical anti-cancer therapeutic method to suppress cancer progression binding with traditional chemotherapy [15, 16]. To further validate the above assumption, we suppressed EGFR expression in AVL9 overexpressed cell by cetuximab treatment (1μM) and performed the rescue experiments. Trans-well assay revealed that decreased EGFR expression could partially inhibit the increased migration caused by AVL9 overexpression in DLD1 cell, indicating that AVL9 promotes CRC cells migration via regulating EGFR expression (Fig. 5A). EGFR, a well-described transmembrane protein, is a member of ERBB receptor tyrosine kinase superfamily which promotes tumor cell migration [17]. Subsequently, GSEA (gene set enrichment analysis) was performed to explore the underlying mechanism of AVL9-mediated migration of CRC cell based on TCGA datasets. Notably, the result revealed that the gene set of Hallmark_ERBB_Targets was obviously enriched in CRC samples with high AVL9 expression, implying that ERBB signaling pathway was positively associated with AVL9 expression based on the CRC GEO datasets (Fig. 5B). These collective findings support that EGFR/ERBB signaling pathway may be essential for AVL9 mediated migration in CRC.

## Discussion

Incidence and mortality of CRC have been shown an increasing trend in China according to the global estimates for cancers worldwide report. Searching for the robust molecular markers is a common focus in facilitating the diagnosis and prognosis of colorectal carcinoma disease [18]. Utility of several genes has been explored that in CRC [19]. AVL9, a novel protein associated with establishing and maintaining epithelial cell polarity, has been reported that the loss of cell polarity acted as a hallmark of epithelial cancers, indicating that epithelial polarity is involved in cancer tumorigenicity and migration [20–26]. However, the specific role and biological function about AVL9 alone in CRC has not been elucidated.

The current study revealed that AVL9 mRNA expression was significantly increased in colorectal carcinoma tissue compared to corresponding non-cancer tissue based on TCGA database. Meanwhile, expression of AVL9 at mRNA level increased gradually from earlier stage to advanced stage of CRC. Consistent with the above bioinformatics, increased protein level of AVL9 expression in CRC tumor tissues than that in the paired normal tissues was observed by IHC staining and AVL9 expression at protein level was positive correlation with stage of CRC. Moreover, we found that higher expression of AVL9 was relevant to M status and stages, unfavorable to prognosis in CRC patients, and there is no relationship between AVL9 and other clinicopathological variables such as gender, age, tumor size, differentiation, T classification and N status. CCK-8 assay and trans-well assay showed that no obvious difference was found on the CRC cell proliferation upon interfering the expression of AVL9, but interestingly, cell migration was significantly influenced, strongly supporting the notion that AVL9 is migration-associated gene. Mechanically, we found that overexpressed AVL9 increased EGFR protein expression while downregulated AVL9 suppressed EGFR protein expression.

Epidermal growth factor receptor (EGFR), a 170-kDa transmembrane glycoprotein composed of an intracellular tyrosine-kinase domain, is one of the anticancer drug targets for colorectal cancer [27]. Recently, EGFR has been reported to be elevated and correlated with advanced tumor stage and increased risk of metastasis in CRC [28–30]. Rescue experiment showed that cetuximab, an EGFR monoclonal antibody, attenuated the effect of upregulated AVL9 on the CRC cell migration promotion. Considering that EGFR is a member of ERBB receptor tyrosine kinase superfamily which promotes tumor cell migration. GSEA analysis suggested that ERBB signaling pathway was positively associated with AVL9 in CRC. These results indicated that EGFR serves as the downstream effector of AVL9 in modulating CRC cell migration.

Furthermore, we also detected EGFR expression at protein level in the aforementioned five kinds of CRC cell lines that derived from five different CRC patients and found no direct correlation between AVL9 and EGFR (data not shown). This may be due to the small size of samples. The future detection should increase the clinical sample amount and maybe obtain an expected result. Similarly, because of distinct gene background in each cell line, we also did not find obvious correlations between AVL9 expression and the ability of migration in these CRC cell lines. EGFR, a well-known cancer promoter factor, facilitates tumor cell proliferation and migration [31–34]. In this regard, it is not clear why AVL9 can upregulate EGFR to enhance cell migration but cannot have an effect on cell proliferation. For this goal, transcriptomic and proteomic analyses for the identification of AVL9 and EGFR downstream co-effectors should be conducted in future.

## Conclusion

Taken together, we demonstrated that AVL9 was upregulated in CRC and it could function as an oncogene in CRC progression. EGFR was regulated by AVL9 and involved in AVL9-mediated cell migration. Our findings provided a new potential biomarker and therapy target for CRC patients.

## Materials and methods

### Tissue microarray and immunohistochemistry staining

The tissue microarray construction (TMA, #HStmA150CS02) containing 83 human CRC cancer specimens and 83 non-cancer specimens was bought from Shanghai Outdo Biotech, Shanghai, China and analyzed by two pathologists in a blinded manner. Detailed clinical information is included, for example, gender, age, tumor size, differentiation, T classification, N status, M status and stages. An anti-AVL9 antibody (ab175108; Abcam, Cambridge, MA, USA) at a dilution of 1:500 was used to detect the expression of AVL9. The classification of ICH staining (immunohistochemical staining) results was as mentioned [35]. The study was approved by the ethics committee of Shanghai East Hospital, Tongji University School of Medicine.

### Cell lines and culture condition

Five kinds of human CRC lines (HCT116, DLD1, RKO, LOVO, SW620) obtained from the Shanghai Cell Bank of the Chinese Academy of Sciences (Shanghai, China), were cultured in Dulbecco’s modified Eagle’s medium ( DMEM; Corning, Inc, Corning, NY, USA) supplemented with 10% FBS (fetal bovine serum) and 1% penicillin/streptomycin (M&C Gene Technology Ltd, Beijing, China). All the cells were incubated in a humidified environment at 37°C with 5% CO_2_.

### siRNA interference, plasmid construction and transfection

The expression of AVL9 in CRC cells were downregulated by transient with two different siRNAs which were synthesized by GenePharma, Shanghai, China. siRNA sense sequences were as follows: siNC (sense 5-UUCUCCGAACGUGUCACGU-dTdT-3), siAVL9-1 (sense 5-GCCACAGUAUUUGGUAUCUdTdT-3), siAVL9-2 (sense 5-GGACUCUUCAUGGCAUCAAdTdT-3). Overexpressed plasmid, a full-length AVL9 cDNA was cloned into pcDNA 3.1 mammalian expression vector, was purchased from Vigene Biosciences, Shandong, China. Following the manufacturer’s instructions, siRNAs or plasmids were transfected using lipofectamine 3000 (Invitrogen, Thermo Fisher Scientific, US). In order to stably overexpressed or knockdown the expression of AVL9, Lentiviral particles (Lv6-AVL9 and Lv6-NC; Lv3-shAVL9 and Lv3-shNC based on siAVL9-1 and siNC sequence) was purchased from Gene Pharma, Shanghai, China. CRC cells stably infected cells were selected by puromycin (2μg/ml) after transducing cells with lentiviral particles.

### CCK-8 assay

Proliferation assay, the cell counting kit-8 (CCK-8) assay, was conducted in accordance with the manufacturer’s instructions. In brief, the appropriate density of CRC cells transfected with siRNA or plasmid were seeded into 96-well plate per cell culture condition above mentioned. Each well was treated with 10ul CCK-8 reagent (Dojindo, Japan) and continued to be cultured for 1.5h. Then, the absorbance value at 450nm was measured spectrophotometrically for five consecutive days. Each experiment was independently performed 3 times in triplicate.

### Cell migration assay

Trans-well assay was performed to measure cell migration using a 24-well trans-well chambers (pore size, 8μm; Costar; Corning, Inc). 5×10^4^ cells counted were cultivated in the upper chamber containing 400μl DMEM free FBS while 800μl DMEM with 10% FBS were supplemented in the bottom chambers. After 24-28h incubation, migrated cells at the bottom of chamber were stained with 0.5% crystal violet and calculated with microscope. Three independent assays with triplicate was performed.

### Wound healing assay

CRC cells stably overexpressing or knocking down AVL9 and corresponding control cells seeded in 6-well plate at 37°C with 5% CO2 were scratched by a sterile 200μL plastic tip (90% confluence). Firstly, rinse off the dead cells with 1× PBS. Then, the cells were cultivated in serum-free DMEM medium for up to 48 h culture. Pictures were taken by the light microscope (Nikon, Japan) with a microscope at 200× magnification. The experiments were performed at least three times.

### Western blot assay

Western blot analysis was carried out as described previously [36]. The Primary antibodies used in this study are mainly: anti-AVL9 (ab175108, Abcam, 1:500), anti-E-cadherin (#3195, Cell Signaling Technology, 1:1000), anti-β-actin (sc-58673, Santa Cruz Biotechnology, 1:500), anti-p53 (#sc-47698, Santa Cruz Biotechnology, 1:500), anti-PTEN (#559600, BD Biosciences, 1:500), anti-EGFR (#4267, Cell Signaling Technology, 1:500), anti-CDK4(#12790, Cell Signaling Technology, 1:800), anti-CDK6 (#14052-1-AP, ProteinTech, 1:500), anti-FAK (#66258-1-Ig, Proteintech, 1:500). Secondary antibodies are as follows: anti-rabbit-DyLight 800 (#SA5–35571, 1:1000, Thermo Fisher Scientific) and anti-mouse-DyLight 800 (#SA5–35521, 1:1000, Thermo Fisher Scientific).

### Datasets and Gene set enrichment analysis (GSEA)

The public data, gene expression at mRNA level in tumor and corresponding non-tumor CRC tissue, was obtained using GEPIA online tools based on TCGA database (http://gepia.cancer-pku.cn/). GSEA analysis was performed to explore biological signaling pathway between the AVL9 high expression group and the AVL9 low expression group. In detail, GSEA analysis was applied using GSEA 4.0.3 (https://www.gsea-msigdb.org/gsea/index.jsp).

### Data accessibility

Gene analysis in TCGA dataset was performed by online tool GEPIA (http://gepia.cancer-pku.cn/).

### Statistical analysis

Statistical analysis for three independent experiments was analyzed by GraphPad Prism 8. The quantitative data was represented as mean ± SD. Difference comparison were analyzed by Student *t* test or one-way ANOVA. Relationships between AVL9 expression and clinicopathological characteristics were analyzed using χ2 test. *P* < 0.05 was served to show statistical significance.

## Data Availability

All data analyzed and generated in this study are included in this published article.

## Abbreviations

CRC: Colorectal carcinoma
NSCLC: Non-small-cell lung cancer
ccRCC: Clear cell renal cell carcinomas
TCGA: The cancer genome atlas
EGFR: Epidermal growth factor receptor
FAK: Focal adhesion kinase
PTEN: Phosphatase And Tensin Homolog
CDK4: Cyclin dependent kinase 4
CDK6: Cyclin dependent kinase 6
mCRC: Metastatic colorectal carcinoma
CTX: Cetuximab
GSEA: Gene set enrichment analysis
TMA: Tissue microarray construction
IHC staining: Immunohistochemical staining
